# Structure Based Design and Molecular Docking Studies for Phosphorylated Tau Inhibitors in Alzheimer’s Disease

**DOI:** 10.3390/cells8030260

**Published:** 2019-03-19

**Authors:** Jangampalli Adi Pradeepkiran, P. Hemachandra Reddy

**Affiliations:** 1 Internal Medicine Department, Texas Tech University Health Sciences Center, 3601 4th Street, Lubbock, TX 79430, USA; pradeep.jangampalli@ttuhsc.edu; 2 Cell Biology & Biochemistry Department, Texas Tech University Health Sciences Center, 3601 4th Street, Lubbock, TX 79430, USA; 3 Pharmacology & Neuroscience Department, Texas Tech University Health Sciences Center, 3601 4th Street, Lubbock, TX 79430, USA; 4 Neurology Department, Texas Tech University Health Sciences Center, 3601 4th Street, Lubbock, TX 79430, USA; 5 Speech, Language and Hearing Sciences Departments, Texas Tech University Health Sciences Center, 3601 4th Street, Lubbock, TX 79430, USA; 6 Garrison Institute on Aging, South West Campus, Texas Tech University Health Sciences Center, 6630 S. Quaker Suite E, Lubbock, TX 79413, USA; 7 Public Health, Texas Tech University Health Sciences Center, 3601 4th Street, Lubbock, TX 79430, USA

**Keywords:** Alzheimer’s disease, p-tau, hyperphosphorylation, pharmacophores, molecular docking

## Abstract

The purpose of our study is to identify phosphorylated tau (p-tau) inhibitors. P-tau has recently received great interest as a potential drug target in Alzheimer’s disease (AD). The continuous failure of Aβ-targeted therapeutics recommends an alternative drug target to treat AD. There is increasing evidence and growing awareness of tau, which plays a central role in AD pathophysiology, including tangles formation, abnormal activation of phosphatases/kinases, leading p-tau aggregation in AD neurons. In the present study, we performed computational pharmacophore models, molecular docking, and simulation studies for p-tau in order to identify hyperphosphorylated sites. We found multiple serine sites that altered the R1/R2 repeats flanking sequences in the tau protein, affecting the microtubule binding ability of tau. The ligand molecules exhibited the p-O ester scaffolds with inhibitory and/or blocking actions against serine residues of p-tau. Our molecular docking results revealed five ligands that showed high docking scores and optimal protein-ligand interactions of p-tau. These five ligands showed the best pharmacokinetic and physicochemical properties, including good absorption, distribution, metabolism, and excretion (ADME) and admetSAR toxicity tests. The p-tau pharmacophore based drug discovery models provide the comprehensive and rapid drug interventions in AD, and tauopathies are expected to be the prospective future therapeutic approach in AD.

## 1. Introduction

Alzheimer’s disease (AD) is a devastating mental illness with an irreversible progressive brain disorder that slowly destroys memory skills and learning abilities. AD is the sixth leading cause of death in the United States [1]. The disease progression and risk factors of AD are not completely understood. During the preclinical stage of AD, people seem to be symptom-free, but toxic changes are taking place in the brain [2]. It seems likely that damage to the brain starts a decade or more before the memory and other cognitive problems appear. AD progression stages vary from mild to severe in middle age people to older persons detected with cognitive tests [3]. The pathophysiology of AD features abnormal accumulation of amyloid beta (Aβ) and phosphorylated tau (P-tau) throughout the brain, which causes healthy neurons to malfunction with synaptic damage and neuronal dysfunction, ultimately leading to neuronal death and cognitive decline in elderly persons [4,5]. 

Normal tau is a highly soluble natively unfolded protein, which contrasts with hyperphosphorylated tau. The microtubule associated protein tau plays an important role in maintaining neuronal structure, stability of microtubules, and neuronal transport [6]. The normal tau becomes aberrant with hyper activation of phosphatases, which leads to paired helical filaments (PHFs) and neurofibrillary tangles (NFTs) in AD brains [7,8,9]. There is an adverse relationship between p-tau and synaptic damage in AD neurons, but precise mechanisms of synaptic damage are not completely understood. 

Several studies revealed that the involvement of tau in synaptic starvation and neurodegeneration is associated with the imbalanced states of phosphatases and kinases in a neuronal cell [10]. The tau is a harbor of kinase dependent residues, nearly 80 serine/threonine/tyrosine, and relays with malfunctions of microtubules in AD state [10,11,12]. The regulation of tau phosphorylation and kinase sites dependent studies were largely inspected in the past in order to understand the significance of p-tau sites and kinase-based expression in AD. Tau phosphorylation sites relay on a number of residues and are the extent of this imbalanced consequence linked to the neurofibrillary tangles formation in AD brains [13]. 

Hyperphosphorylated forms of tau protein are the main component of PHFs of NFTs in the brain of AD patients. It has been well demonstrated that regions of tau six-residue segments, namely PHF6 (VQIVYK) and PHF6 (VQIINK), can form tau PHF aggregation in AD [14]. Apart from the PHF6, some other residue sites like Ser285, Ser289, Ser293, Ser305, and Tyr310, located near the C-terminal of the PHF6 sequences, play key roles in the phosphorylation of tau [15]. Between the six-residue segment, four other possible tau phosphorylation sites have also been identified as important in AD. These four sites of tau are involved in AD by misbalancing the kinases metabolism by activating phosphorylase kinase (PK), casein kinase 1 δ (CK1 δ), and/or glycogen synthase kinase-3 (GSK-3) [16].

Serine specific tau phosphorylation has been reported to be involved in tau pathology of AD [17]. The identification and validation of p-tau based serine targeted site-specific kinase dependent inhibitors are considerably the best therapeutic appeals in AD and other tauopathies models in helping to find new therapeutics.

The therapeutic strategy for p-tau phosphorylation in AD and other tauopathies based upon its inhibition is interesting. However, kinases are a difficult class of drug targets, as they trigger the multiple residues of protein. Therefore, when designing the selective molecules, it is quite challenging to avoid off-target toxicity. The current drug discovery approaches, transformed by speeding the rational ligand screening applications and pharmacokinetic studies, laid a new flat form to discover the small molecules against p-tau including AD. From the last decade to present date, the status in neuro-therapeutic drug development has been less successful due to unbiased modes of drug interactions attributed to failed results [18]. This current challenging situation makes considering the rational therapeutic development for AD and other tauopathy diseases an urgent matter [19,20]. 

Computational methods are, in principle, analogous to high-throughput screening in that both target and ligand structure information is required. The extending features of 3D ligand based pharmacophore screening and pharmacokinetic studies improve the lead molecules selection with ligand efficacy [21,22]. 

The present study aimed to identify and validate the serine tau specific inhibitors on ubiquitously expressed p-tau residues in AD and other tauopathies. We used the following approaches to determine the efficacy of the p-tau ligand inhibitor molecules: (1) homology modeling; (2) 3D refine; (3) simulations; (4) molecular dynamic simulations; (5) 3D pharmacophore; (6) molecular docking; and (7) ADME (absorption, distribution, metabolism, and excretion) and admetSAR studies. These are important approaches to determining p-tau ligand inhibitors.

## 2. Methods

### 2.1. Homology Modeling

Due to the non-availability of the crystal structure of tau in mouse models, we constructed a protein 3D structure using MODELLER 9.13v (Ben Webb at the Departments of Biopharmaceutical Sciences and Pharmaceutical Chemistry, and California Institute for Quantitative Biomedical Research, Mission Bay Byers Hall, University of California San Francisco, San Francisco, CA 94143, USA) [23]. In order to model the protein, we ran the BLAST-P against protein data bank (PDB) results, which revealed a perfect reliable template, i.e., the crystal structure of a microtubule-associated protein human tau (PDB: 2MZ7) that shared 90% query coverage and 80% identity with 1e-53 *E*-Value. The aligned 2D results of the blast showed a high-level sequence similarity between target and template. Based on alignment, we chose the template structure for modeling and built 100 models using the comparative modeling method. Furthermore, homology 3D modeled tau was evaluated for its stereo-chemical quality check, and further analysis was performed using RAMPAGE server (Department of Biochemistry, University of Cambridge, Cambridge, UK) [24] and ProSA analysis (Center of Applied Molecular Engineering, Department of Biosciences University of Salzburg, Austria) [25].

### 2.2. 3-Drefine

After the initial quality check of the modeled protein tau analysis, we ran the 3D refine with an interactive web server for consistent and computationally efficient protein structure stability with statistical and visual analysis algorithms. The server-programmed algorithms used the optimization of the hydrogen bonding network with the combined atomic-level energy. This refinement procedure was composed of composite physics and knowledge based force fields methods to evaluate the blind critical assessment of structure prediction (CASP) experiment and gave the final structure in both global and local quality measures [26]. The stable 3D structure was considered the best model for molecular dynamics and molecular docking studies and therefore the model we chose to use as the final 3D refine model for further analysis.

### 2.3. Simulations

The molecular dynamics (MD) simulations in a biological system is considered to be an important step for the theoretical research application. The tau protein simulations were run with a protein dynamics is simulated using a CABS coarse-grained protein model CABS-flex server, utilizing the high-resolution coarse-grained representation of a protein chain in which a single protein residue was represented by up to four atoms (the Cα and Cβ atoms and two virtual pseudo-atoms) [27]. The tau simulation results displayed the residue fluctuations of tau with molecular dynamics simulation data. The final stable structure of tau was taken for further analysis and studies.

### 2.4. Molecular Dynamics

MDWeb is powered by a set of BioMoby Molecular Dynamics Web-Services (MDMoby) (NBD Nostrum Biodiscovery, Barcelona, Spain), used for the analysis and molecular dynamic simulations of refined tau protein. The web portal MDWeb provided a friendly environment to set up new systems, run test simulations, and perform analysis within a guided interface. The tau protein set up files for molecular dynamic simulation on MDWeb could be prepared for Nanoscale Molecular Dynamics (NAMD) and the Charmm2 force fields methods, and the tau MD simulation results analysis could be carried out using any standard trajectory read format root-mean-square deviation (RMSD) with applicable tools [28].

### 2.5. Active Site Prediction

The prediction of functional sites included ligand binding sites, functional sites of small molecules that interact with a protein and modulate the function of the protein. After the 3D refine, the prediction of the tau active site is an important molecular feature for the “small-molecule pocket” of tau detection for ligand binding probabilities and biological functions of proteins. The Fpocket server (Axel Bidon-Chanal and Javier Luque team from the University of Barcelona, Barcelona, Spain) provided pocket detection, thus we tested the tau 3D structural domains and active site residues of tau with a computed atlas of surface topography of proteins, then identified the best ranked pockets from the holo and Apo proteins with the Fpocket server [29].

### 2.6. Pharmacophore Generation

The modeled structure of the tau protein is a ligand free structure, hence we selected a Ser/Thr specific ligand (7-Phosphonomethyl-naphthalene-1-carboxylic acid) to be used for the construction of the ligand-based pharmacophore models. A ligand scout (LS) tool permitted the automated construction and visualization of the 3D pharmacophore based on the structural knowledge of the macromolecules/ligand complexes [30]. The LS algorithm extracted the chemical features in terms of electrostatic topographies, such as hydrogen bond donors and acceptors as directed vectors. The p-tau 3D pharmacophore features such as positive, negative, and ionizable portions of the receptor molecule are represented by spheres of colors with different combinations depicted in the LS tool. The best fitted pharmacophore ligands were set up for the molecular docking studies in molecular operating environment (MOE) [31].

### 2.7. Molecular Docking

Before going to docking, we prepared the tau protein 3D coordinates by loading the homology modeled tau in MOE and removing the water molecules. Then, we added the polar hydrogens to the protein. Then, the protein molecule with protonate 3D was done with a temperature of 300 K, a salt concentration of 0.1, and a pH of 7.0 within a solvated environment to start the 3D protonation. Next, the protein structure underwent energy minimization with a force field Merck molecular force field 94x (MMFF94x) and a root-mean-square (RMS) gradient of 0.05. During the energy minimization, conformations of tau were subjected to molecular dynamic simulations with a constant temperature. An active site was defined on the tau molecule for the ligand binding interactions by selecting the core residues of tau, i.e., Ser23, Ser27, Ser39, and Tyr44, and then computing the simulation by set up dock. All the tau docking confirmations and ligand binding interactions were read in the ligand interaction panel. 

### 2.8. ADMET Prediction

Any drug development process involves the assessment of basic ligand features like absorption, distribution, metabolism, elimination, toxicity, and drug-likeness properties in order to explore the ligand properties. The physicochemical behaviors of ligands were used to analyze the pharmacokinetic properties of ligand molecules in a biological environment. The in silico ADME pharmacokinetic properties were analyzed by the FAFdrugs4 [32], and the admetSAR [33] property explorer validated the ligand properties of toxicity in biological activity. The in silico prediction methods constituted a valid alternative to experiments and provided a global appraisal of the pharmacokinetics profile of small molecules to develop rational drug development. 

Overall, analysis of the computational screening, p-tau pharmacophore models, and molecular dynamic simulations and docking studies were utilized to find the best non-toxic p-tau AD therapeutics in drug discovery. 

## 3. Results and Discussion

### 3.1. Homology Models Validation

**Figure 1:**
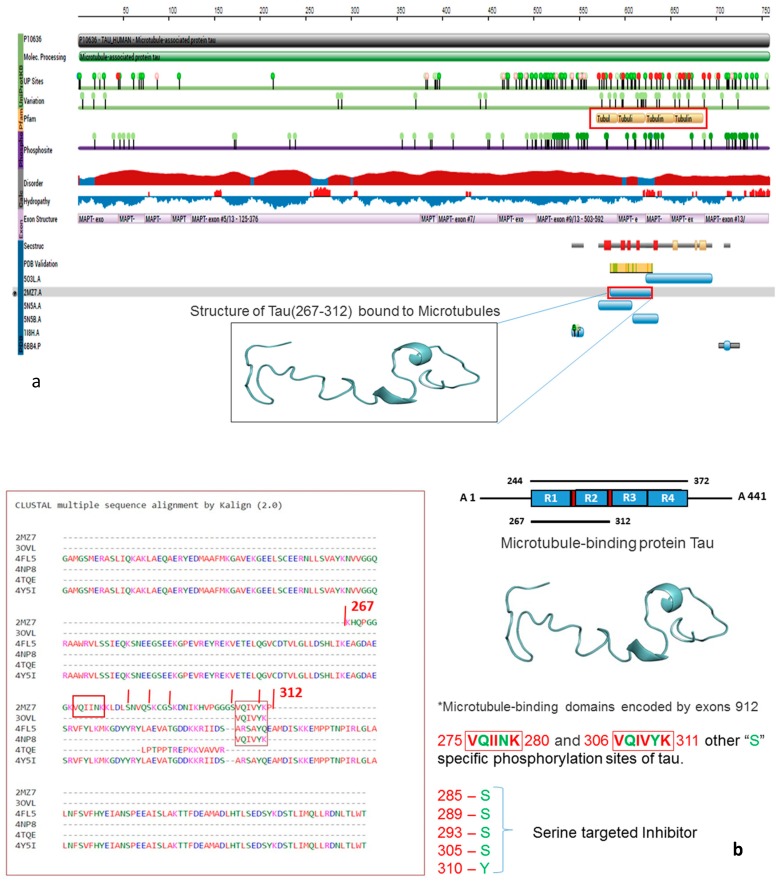

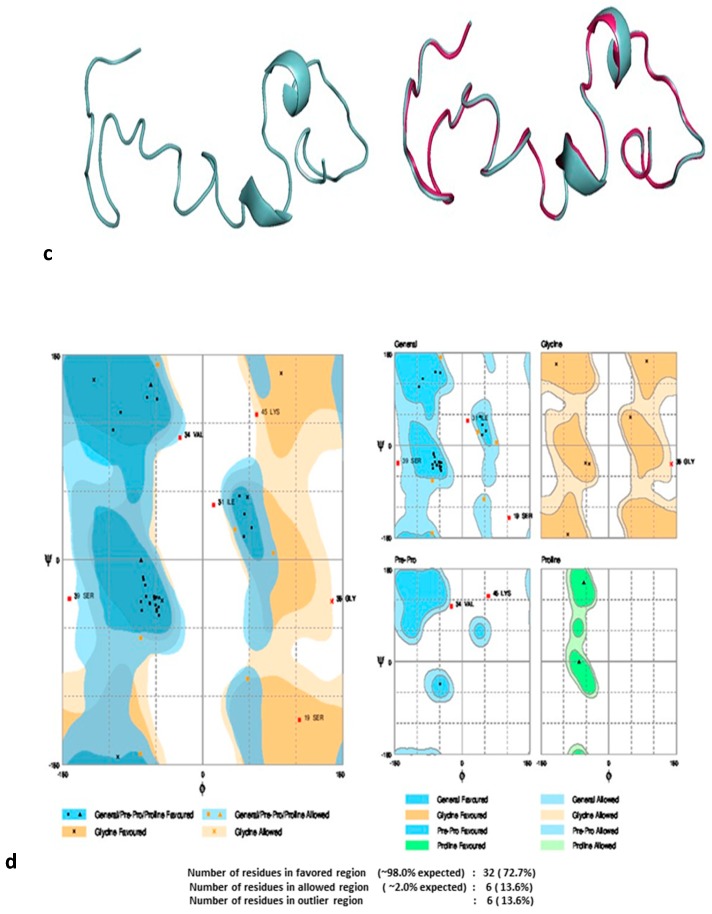
(**a**) The functional domains of the tau gene displays the phosphorylation sites and variations across the six matured tau isoforms, as well as a zoomed view of microtubule associated gene tau location of residues boundaries; (**b**) multiple sequence alignment of tau six isoforms highlighted on complete gene microtubule associated gene tau (MAPT) and R1 and R2 localization of tau with common functional domains of tau isoforms, i.e., the VQIINK and the VQIVYK of the tau gene domains contain R2 and R3 with SER/TYR/THR residues, which are involved in the phosphorylation active sites; (**c**) the homology modeled tau protein (cyan colored) structure superimposed with the 3D structure of human tau in red; (**d**) Evaluation of steriochemical quality of modeled tau protein with RAMPAGE analysis (Ramachandran plot) showing 98.0% of residues were found in the favored region (thick blue background with dark square and triangle dots) general pre-pro/proline favored, general pre-pro/proline allowed region (light blue background with yellow square and triangle dots) 2.0% in the glycine favored (dark yellow background with squared dark triangle) glycine allowed (light yellow back ground with dark squared triangle), and none in the outlier regions (white). Four different portions were displayed in (right side) General, Glycine, Pre-proline and Proline amino acids distribution based up the ψ and ϕ angles.

The non-availability of the tau protein structure in mouse models required us to build a model based on template based protein modeling (TBM) using MODELLAR. Due to the lack of 3D crystal structures for the tau protein, we built 100 models with MODELLAR and selected a low discrete optimized protein energy (DOPE) −1255.61121 with 518.21741 kcal/mol energy. The seventeenth model was considered to be the most reliable model for further validations and molecular docking studies. The modeled tau protein evaluations were done with the SAVES server, which showed the best consistency with RAMPAGE (Ramachandran plot) results. A number of residues were found in the favored region (~98.0%), residues among 32 with 99.0%, and additional allowed regions were ~2.0% with six residues (1.0%). We identified no residues located at disallowed regions, proving that the comparative modeling of the tau protein 3D structure was decent based on the knowledge-based computational approaches (Figure 1). 

**Figure 2:**
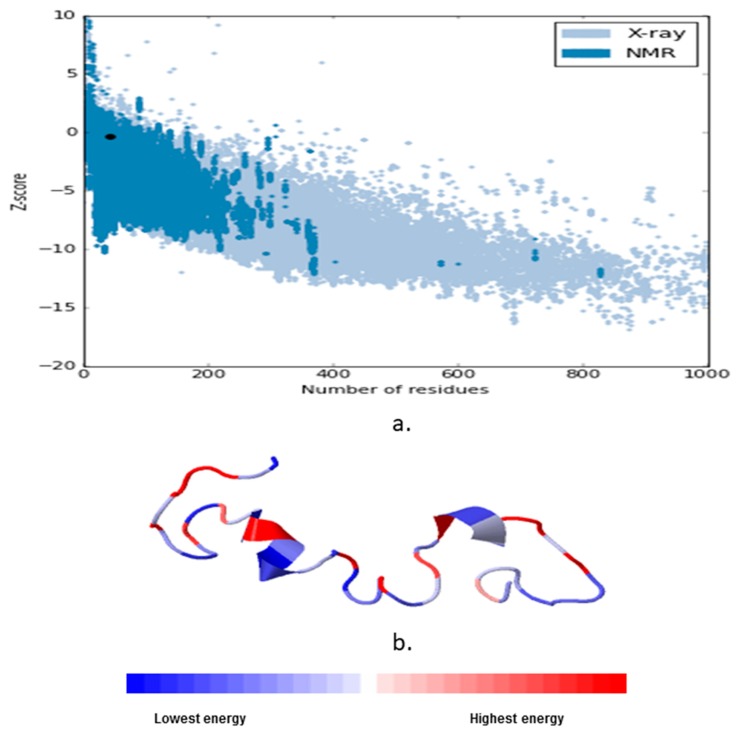
(**a**) The protein structure analysis (ProSA)-web for validation of the experimental protein structures, structure prediction, and modeling. The quality scores of the protein homology modeled tau showed good quality with a Z-score −0.39 with dark dots indicating real experimental quality such as NMR and X-ray crystallography; (**b**) ProSA-web visualized the 3D structure of the modeled, tau protein residues are colored from blue to red in order of increasing residue energy. The tau modeled residue energy distribution was entirely below the zero base line, consistent with the residue number.

We further cross-checked the protein quality assessment of tau with structural analysis (ProSA) and web based interactive analysis, which specified the errors in the 3D structures of the system generated proteins. The results of the ProSA analysis of tau displayed the best consistency with −0.39 Z-score nearer to the NMR 3D models, evidenced by the dark dot (modeled tau protein) position in the scatter plot (Figure 2a). The tau protein energy stabilizations of residues are colored from blue to red in order to display the increasing residue energy and representing the most negative (attractive) and the most positive (repulsive) parts of the modeled tau protein [34] (Figure 2b). 

The tau 3D refinement analysis displayed the qualities of the refined models with global distance test total score (GDT-TS), global distance test high accuracy (GDT-HA), root-mean-square deviation (RMSD), and pair-wise distance-dependent atomic potential functions with the values side-chain orientation-dependent energy (RWplus) and structure validation for macromolecular crystallography (MolProbity). Of the top five tau protein models, model 1 had the best results, with a high 3D refine score of 4716.46, which indicated a better quality model, along with a higher GDT-TS of 1.0000, GDT-HA of 1.0000, RMSD of 0.135, and a low MolProbity of −3230.220671. Therefore, we chose the tau model 1 as the most reliable model to satisfy all the relative values for further analysis (Table 1).

### 3.2. Molecular Dynamics Simulations

Molecular dynamics simulations of experimental validation of protein folding were required to obtain structural validation with sufficient temporal resolution for 3D folding for the protein function. The molecular simulations offered a high resolution of special and temporal data regarding protein folding possibilities, trajectories, and potential energy validations of proteins [35]. The all-atom molecular dynamics simulations (MDS) are the gold standard in simulating protein dynamics. The various simulation methods have been validated in many cases for biologically relevant timescales. For the present study, we used the CABS-flex server to simulate the 3D model of the tau protein with efficient constraints to the all-atom molecular dynamics algorithm, a classical simulation approach for proteins. The clustering of the tau protein models from one to twelve displayed a separation of a set of protein models from the groups to form highly fluctuated models with final stable structures (Table 2).

**Figure 3:**
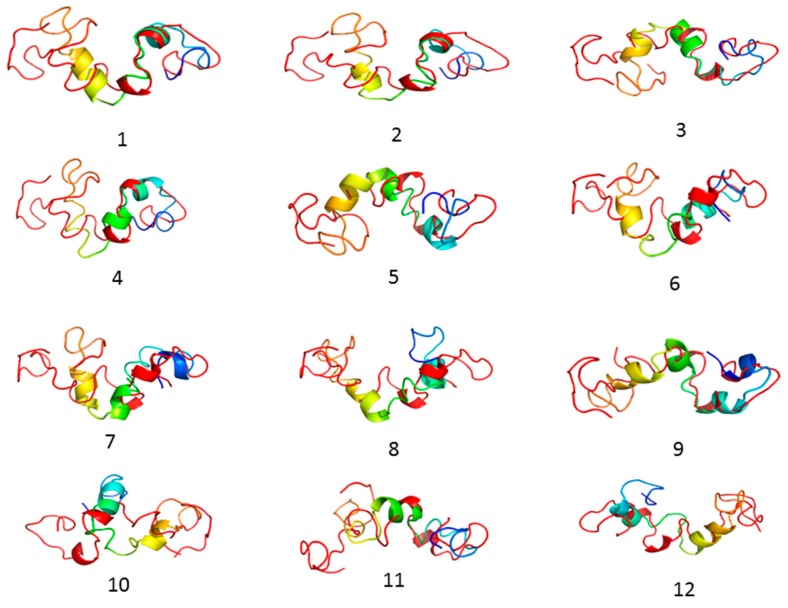
Tau protein 3D simulated structures results displaying the 12 models that employed restrained backbone flexibility during energy minimization. Such an iterative scheme is effective in escaping any local minima and moving closer to the native structure.

The RMSD and GDT_TS values for the 12 protein models displayed in Table 3, the Cα RMSD between predicted models clearly shows that model 1 was more stable with lower RMSD of 6.94 compared to the remaining models (Table 3). All 12 simulated protein models and fluctuated protein models are displayed in Figure 3. 

**Figure 4:**
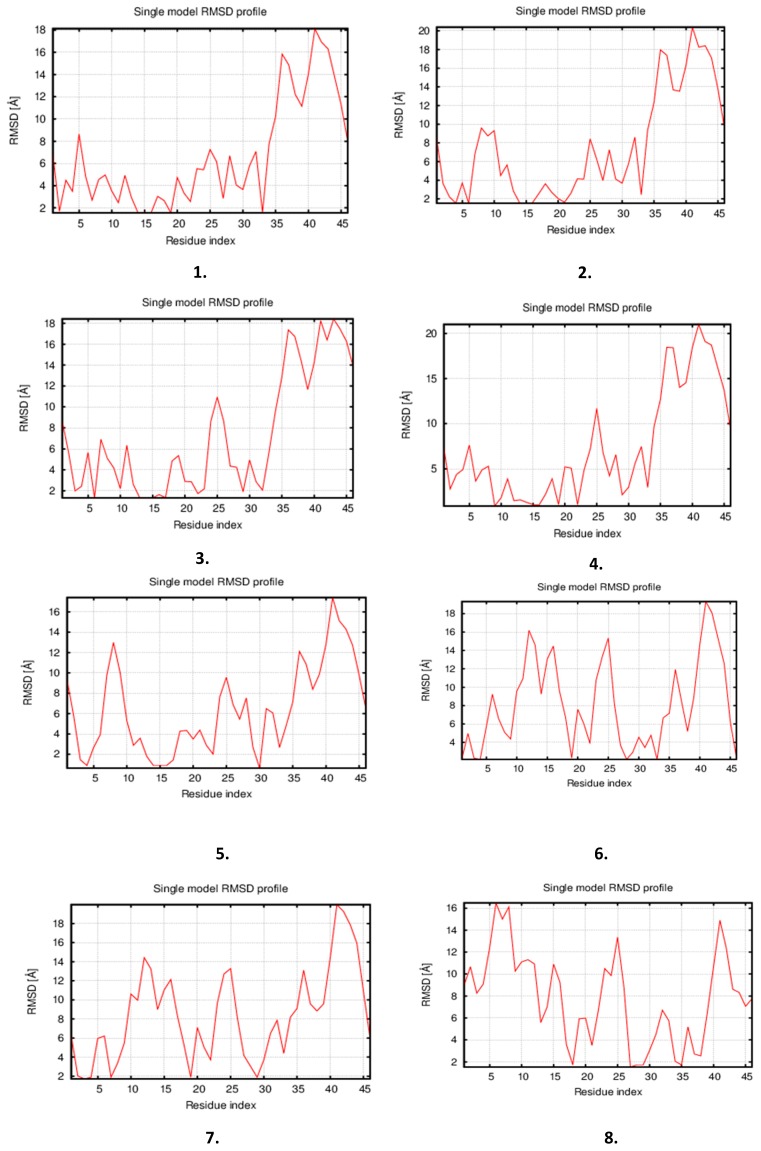

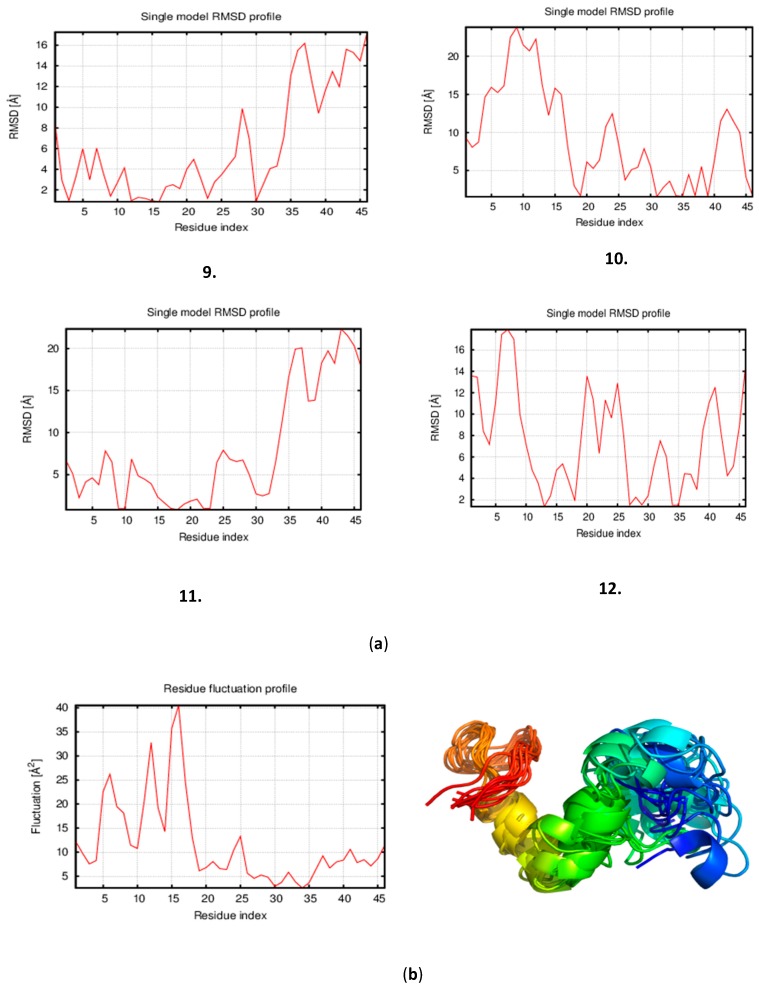
(**a**) Tau protein 3D simulated structure models displayed with 12 models of RMSD fluctuations and flexibility during energy minimization, displayed in terms of the residue-fluctuation profile index of protein models. The lowest RMSD graph displayed was considered as the final refined tau model; (**b**) The final refined stable tau protein model displayed, superimposed with 12 simulated tau protein models.

All 12 simulated protein models and fluctuated residue profile plots are displayed in Figure 4a. The final 3D simulated structures were superimposed models, and the final residue fluctuated plot is displayed in (Figure 4b). 

**Figure 5:**
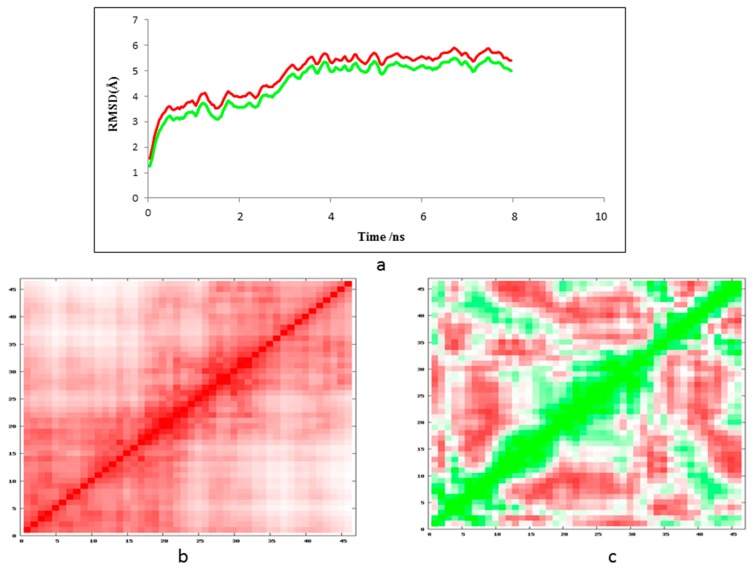
(**a**) MDWeb results of tau protein. Protein backbone RMSD graph for the modeled tau (green) and template tau (red) molecular dynamics (MD); (**b**,**c**) protein tau αC atom root mean square fluctuation (RMSF) values (in Å Angstroms) per residue square matrix graph for the tau before dynamics (red) and after MD dynamics (green).

The final refined model of the tau was set up for the molecular dynamics simulations through MDWeb. The MDWeb results of tau implemented in hypertext preprocessor (PHP) and MySQL provided a graphical user interface (GUI) by utilizing the NAMD2 package. The NAMD2 full dynamics picked a CHARMM 27 force field with three components defining the potential energy of a molecular system as a function of atomic coordinates, atom types, and parameter sets that fit the equations to experimental data. The CHARMM 27 force field maintained constant temperature dynamics via Langevin dynamics and constant pressure dynamics via Nose-Hoover Langevin piston. The MD simulations bond vibrations involving hydrogen atoms was used to maintain all bonds involving hydrogen atoms at their equilibrium values maintained SHAKE algorithm. It provides users with a personal workspace where intermediate data, trajectories, and analysis results can be downloaded. The tau protein residue fluctuations profile showed relative propensities of the backbone protein residues to deviate from an average dynamics (trajectory) structure from the dynamic state to the native structure after the molecular dynamic simulations (Figure 5). 

### 3.3. Ligand Binding Site

**Figure 6:**
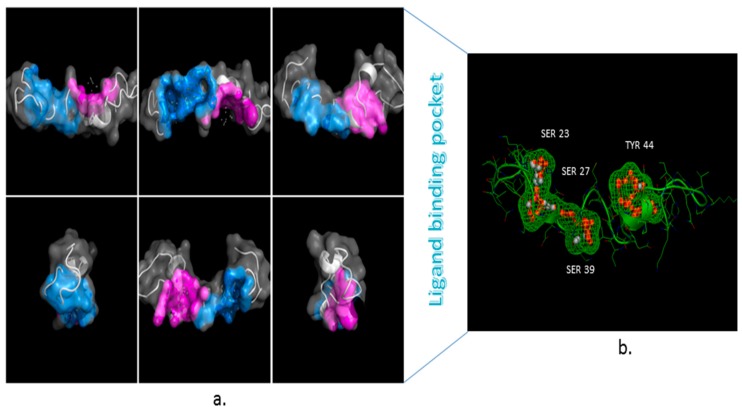
(**a**) Tau Fpocket binding site characteristics, including pocket shape, nature of residues, and interaction profiles on molecular dynamics trajectories protein surface images to detect highly conserved domains; (**b**) Tau protein cavity zoom view of two conserved domains with mesh of Fpocket-predicted Ser and Tyr sites.

Fpocket results identified hits—key residues of the tau protein that bind at ligand specificity with scoring functions and are essential leads for drug discovery research. The Fpocket server relies on a simple scoring function algorithm and identified the binding residues Ser23 (S285), Ser27 (S289), Ser39 (S293), and Tyr44 (Y310) as they pertained to the functional properties of tau. The p-ligand binding pockets within the best three ranked pockets from the holo and Apo proteins identified the two domains reserved for the functional residues like Ser and Tyr (Figure 6). 

### 3.4. Ligand Screening

The best refined stable model of tau from the MDS results was used for further docking studies. The tau protein molecular structure plays an important role in binding to microtubules with different residue combinations. Thus, we hypothesized that four binding residues, Ser23 (S285), Ser27 (S289), Ser39 (S293), and Tyr44 (Y310), pertained to the hyperphosphorylation in AD. We identified no existing ligands in the human tau crystal structure PDB: 2MZ7, hence the selection of the ligand for docking studies was a crucial part. Based on literary evidence, we searched for Serine/threonine-protein phosphatase inhibitors from the Binding DB database [36]. Based on the evidence and ligand properties of molecules, we chose the most promising inhibitor, 7-Phosphonomethyl-naphthalene-1-carboxylic acid (CHEMBL123495), as the template ligand for further pharmacophore studies. This ligand molecule has a carbon-phosphorus bond in place of the scissile P-O ester bond of the natural substrates of phosphatases. As a result of this replacement, phosphonic acids show higher pKa values. The phosphonothioic acids are non-hydrolysable under normal physiological conditions with maximum inhibitory properties. The ligand CHEMBL123495 was trained with ligand scout algorithms to fulfill the screening process. The ligand scout screened the best possible ligand molecules from the databases of semi-synthetic and natural compound libraries like NATx, which contained a total of 436 molecules and saved ligand molecules with spatial data file (SDF) spreadsheets for further docking studies.

### 3.5. Pharmacophore Development

**Figure 7:**
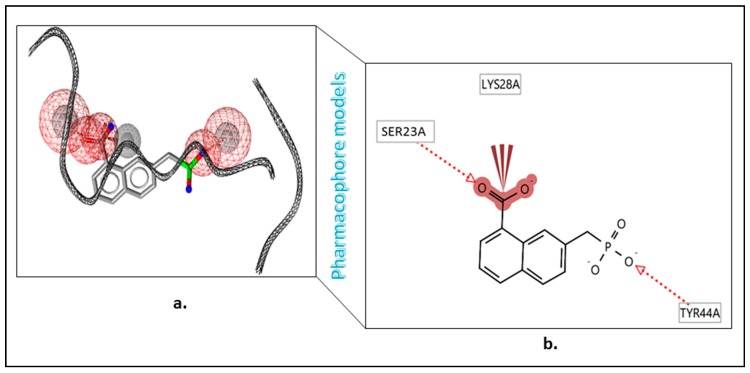
(**a**) Tau pharmacophore model aligned with most active compound-7 Phosphonomethyl-naphthalene-1-carboxylic acid (CHEMBL123495); (**b**) interactions between native ligand and target site amino acid residues with hydrophobic interactions shown as acceptor interactions in red.

The 3D pharmacophore features comprised of chemical features included hydrogen bond acceptors (HBA) (red), hydrogen bond donors (green), and hydrophobic groups (yellow), as well as mechanically generated excluded volumes that are represented in gray [37]. The molecular features of the p-tau model have only two HBAs (Figure 7a). Furthermore, the program mechanically produced many excluded volumes (gray) within the model. The HBA description indicated one phosphate group of the ligand with Tyr44 and one water molecule from the Ser23, respectively (Figure 7b). The tau pharmacophore model mechanically produced by the LS tool was comprised of one feature—five HBAs (red) (Figure 7a). Spheres within 5 Å distance from the inhibitor were generated. Furthermore, the program mechanically produced many excluded volumes (grey) within the model. The HBA description indicated two amino groups of the ligand from the Ser23 (S285) and Tyr44 (Y310), respectively (Figure 7b).

The HBA sphere had a 2.5 radius, θ: 95.27, and φ: 0.02 (coordinates X: −20.85, Y: 86.42, Z: 13.58). The positive ionizable sphere had a 1.3 radius, θ: 76.21, and φ: 0.42 (coordinates X: −19.25, Y: 67.75, Z: 32.25). The hydrophobic sphere had a 1.0 radius, θ: 72.59, and φ: 0.12 (coordinates X: −12.25, Y: 61.32, Z: 38.68). The hydrophobic features were set within a salt-forming anion of phosphoric acid of the ligand. Within the test database, we kept the ligand (CHEMBL123495) present in complex structures. The hydrophobic features were set within the phosphate in an inorganic chemical group of the ligand. Within the test database, we kept the ligand (CHEMBL123495) present in complex structures. First, the ligand CHEMBL123495 was extracted. Then, hydrogen atoms were added and energy minimized by the LS. The screening analysis compounds were properly plotted by the generated pharmacophore models and confirmed the legality of our pharmacophore model, which was subsequently used for the screening of enormous databases. This was our preferred method in drug discovery for finding the nascent target molecules compared to the routine conventional approaches [38]. Then, all the ligand molecules of the NATx library were subjected to training with the pharmacophore features using the LigandScout 4.2v software (Inte:Ligand LigandScout Knime Extensions, Vienna, Austria, Europe), which resulted in 436 ligands with possible lead topographies and supramolecular chemical features of ligands, only 154 of which possessed the best template ligand characteristics of p-tau inhibition.

### 3.6. Molecular Docking

**Figure 8:**
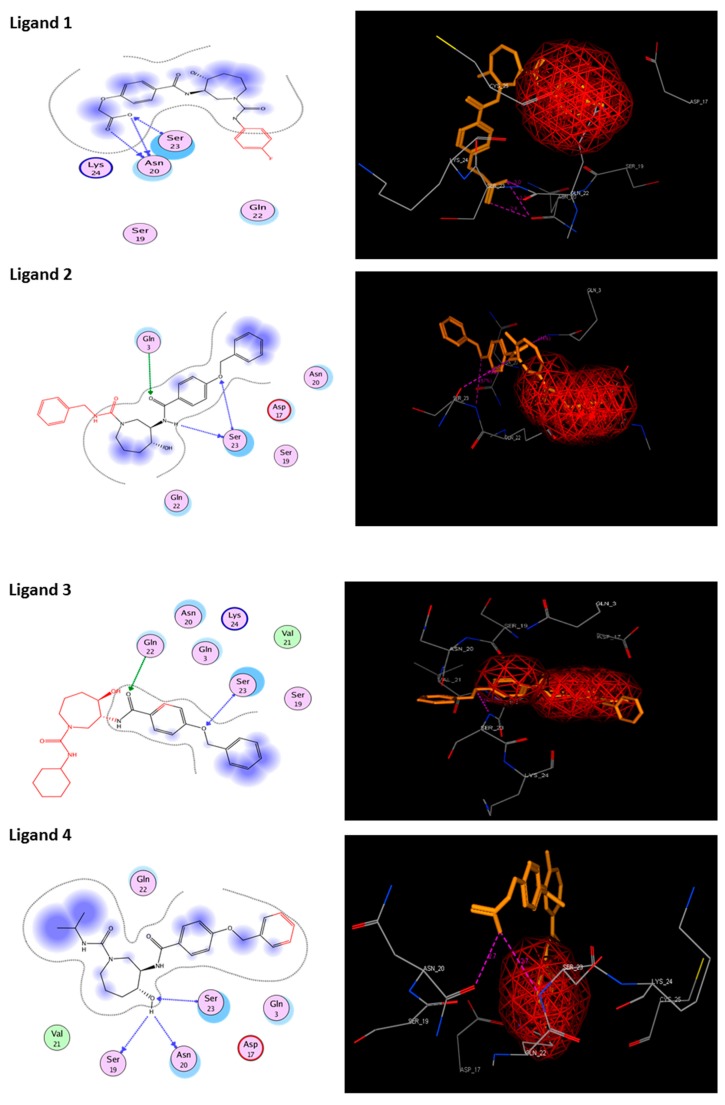

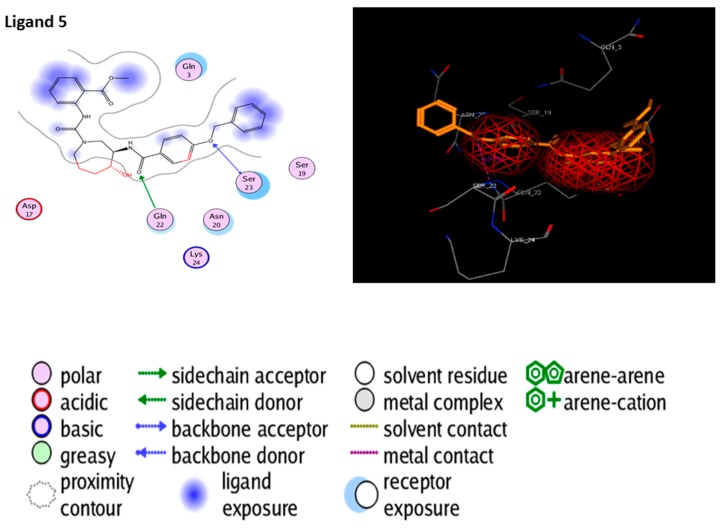
1D-3D ligand interaction diagrams for the top five ligand molecular poses of docking protein- ligand interaction analysis in MOE, along with docking key legends.

Molecular docking studies proceeded with the database of 154 molecules built by MOE and the defined core pharmacophore features of NATx. The MOE docking interactions were analyzed by London ΔG force and ΔE interaction energies, and parameters were set with default algorithms for docking. The p-tau protein-ligand complex was evaluated by measuring various polar and non-polar interactions, such as H-bonding interactions, electrostatic interactions, van der Waal’s interactions, and hydrophobic interactions for each ligand. The lowest docking score (11.39 kcal/mol, ligand 1) was confirmed as the best docking score and so on, with the remaining four lowest docking scores of the remaining ligands visualized through molecular docking interactions. The top five ligand docking results demonstrated the ligand binding energies such as (1–5) molecules −110.32, −110.43, −100.66, −123.36, and −111.03 kcal/mol binding energies with −11.39, −10.73, −10.45, −10.39, and −10.10 docking scores, respectively. The complete docking results displayed the protein–ligand interactions such as hydrogen bonding, bond angles, bond lengths, and type of bonding. All the protein-ligand molecular interactions were analyzed and are illustrated in (Table 4). The docking position and protein-ligand interaction maps of 1D and 3D for the top five molecules are depicted in Figure 8. The docking scores, ligand interfaces, and hydrogen bonding interactions were extended features indicating that the top five ligand leads were more specific inhibitors at serine sites of phosphor-tau. These Ser 285 specific sites are potentially involved in AD brains and have been reported in previous studies [16]. These ligands definitely target CK1δ, GSK-3β, and phosphorylase kinase by inhibiting the hyper-phosphorylation in AD [8]. Further validations for small molecule tests such as ADME, toxicity, and blood brain barrier (BBB) are challenging tasks for drug delivery to the central nervous system (CNS) in modern medicine. The computer-based in silico predictions for BBB done with lazar, a modular predictive tool [39], described the BBB properties of all ligands, which showed positive penetrating properties (Table 5). All these properties were examined before the real experimental validations such as in vitro and in vivo studies of p-tau inhibition in AD and tauopathy diseases.

### 3.7. In Silico ADMET Analysis

**Figure 9:**
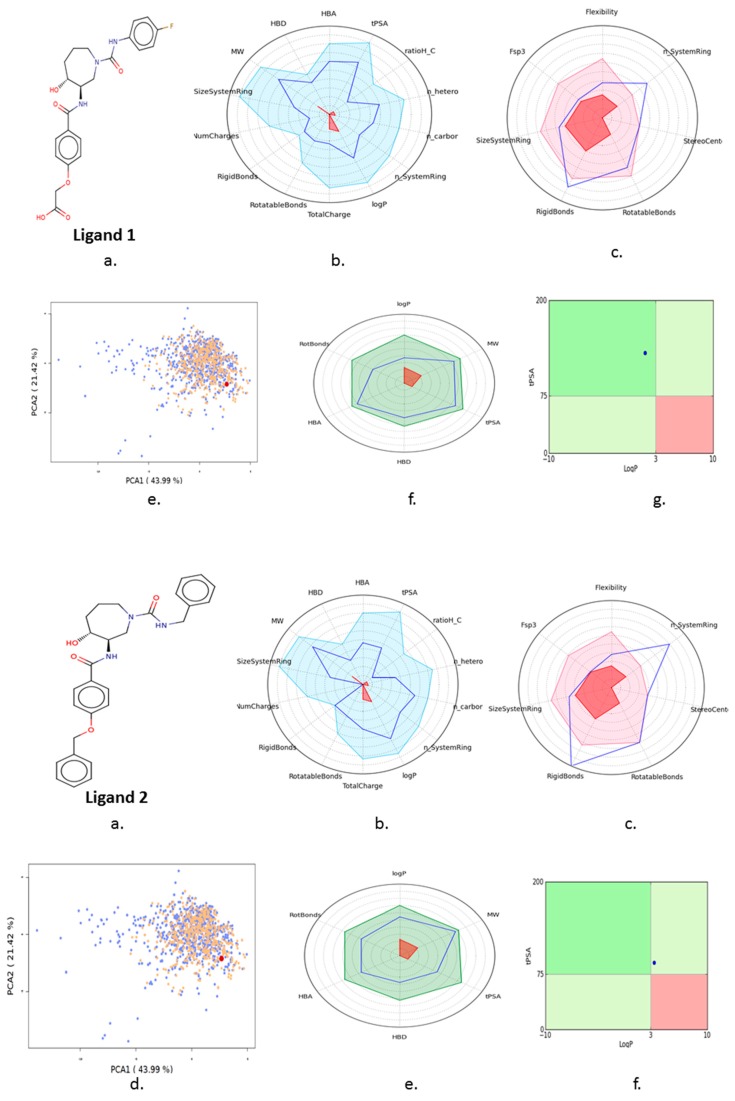

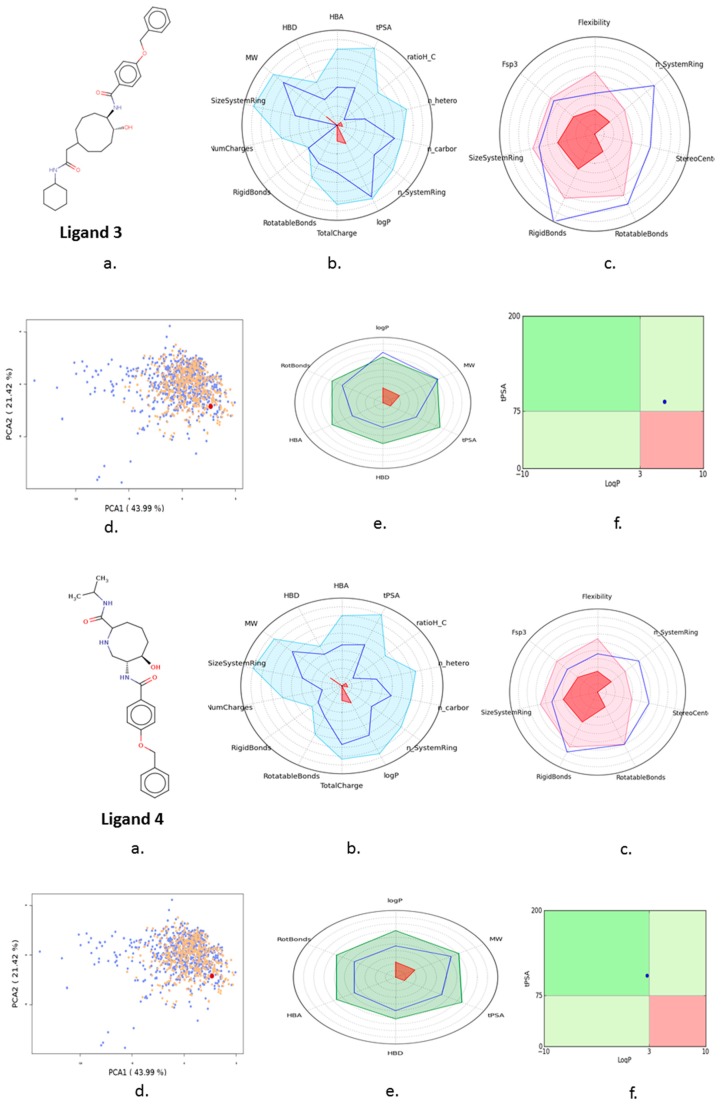

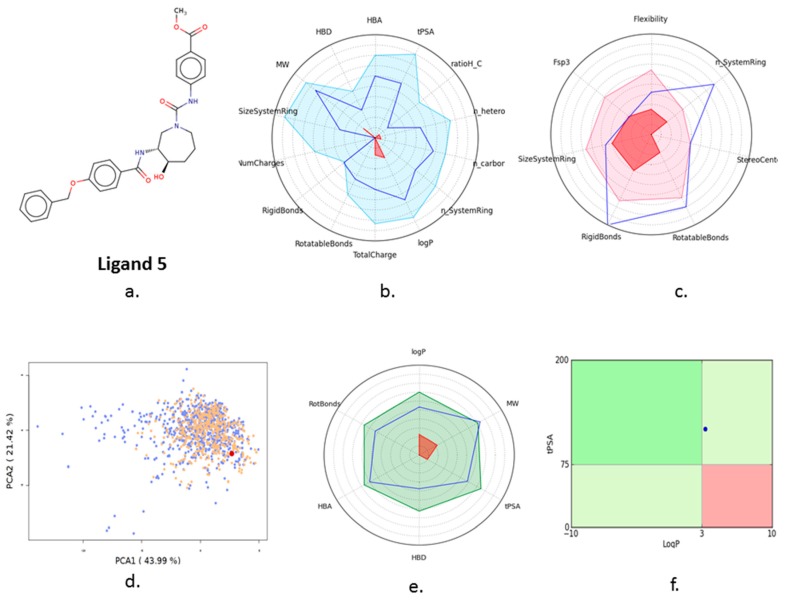
FAF-Drugs4 results for the top five ligand molecules and their respective ADME properties such as: (**a**) 2D structure of each ligand atoms; (**b**) physicochemical filter positioning of all five ligands; (**c**) compound complexity of all five ligands; (**d**) oral property space of all five ligands; (**e**) oral absorption estimation of all five ligands (**f**) Pfizer 3/75 rule positioning of all five ligands.

All of the best five docking ligand molecules were screened for pharmacokinetic properties such as ADMET to investigate the relationships between ligands’ physicochemical properties, potency, and the ADMET profile of small molecules. In order to crosscheck all five ligand molecules, we performed computational screenings such as FAF-Drugs4 and admetSAR analysis to find the solubility and permeable of the ligand molecules in order to use them for experimental assays and to reach their site of action in an accurate drug ability. The computational algorithms were essentially developed based on the molecular properties of chemical boundaries of ligand molecular properties, such as molecular weight, Mol *Log*-P, H-bond donors, and H-bond acceptors (MWT_500, logP_5, H-bond donors_5, and H-bond acceptors_10). The computational screening of ligand molecules, physicochemical properties, and molecular behavior of the compounds included extended features such as the topological polar surface area (TPSA), which was investigated by the analysis of rotatable bonds, a measure of molecular complexity, and the number of stereocenters. The molecular complexity of the five ligands could be measured by the number of rings and aromatic rings, the fraction of carbons that were sp3 hybridized (Fsp3), or the number of stereocenter properties, which were all computed by FAF-Drugs4 (Figure 9). 

The ligand toxicity is a measurement of the degree to which a substance can damage an organism or substructures of the organism. The most significant reasons for late-stage drug development failure are ligand toxicity in late-stage identification during the in vivo drug toxicities and drug development. The in silico ligand toxicity and biological property predictions are fast and more reliable approaches to take before further investigating experimental validations such as in vitro and in vivo tests. All five ligand molecules’ properties were screened with the admetSAR server, and results conveyed that all five ligands were non-toxic and passed the AMES carcinogens may be identified via this test. All five p-tau inhibitor molecules obeyed the Lipinski rule of five and ADMET properties with biological possible activity. Therefore, these p-tau ligand inhibitors are most suitable for further drug discovery approaches. These p-tau inhibitors molecules may possess the best optimal pharmacokinetic properties by inhibiting the hyperphosphorylation of tau in AD and other tauopathies diseases (Table 6).

## 4. Conclusions and Future Directions

In this study, we selected tau as a target protein, and reported five highly selective, site specific Serine protein kinase based tau inhibitors. Our results exhibited the top five ligand molecules, showing the strong binding affinity with the tau receptor site through molecular docking energies and protein-ligand molecular interactions such as hydrogen bonding and ionic interactions with receptor tau molecules. 3D pharmacophore drug discovery methods that produced the desired structural features of lead molecules like HBA, HBD, hydrophobic, and supramolecular features were used for selecting the best potential ligand leads from a large dataset. The in silico ADME, admetSAR, and BBB results indicated that the top five lead molecules (Ligands 1–5) were the potential inhibitors and non-toxic attributes to clinical safety concerns of in p-tau therapeutics in AD and tauopathies models. 

We hope these inhibitors act as effective therapeutic targets in the future; furthermore, in vitro (cell culture) and in vivo (animal model) studies are required to evaluate the prospective drug development in AD and other tauopathies. In the future, we will test these five ligands (phosphorylated tau inhibitors) using cell culture studies and transgenic tau mouse models of AD and other tauopathies studies. As mentioned above, our future studies of phosphorylated tau inhibitors will provide new information about the efficacies of five ligands, not only for AD but also for other tauopathies. Our proposed studies are serine 285 based phosphorylated tau targeting GSK3α, GSK-3β phosphokinases for AD and other tauopathies. 

## Figures and Tables

**Table 1:** Protein dynamics is simulated using a CABS coarse-grained protein model (CABS-flex) results of tau, top five protein molecular dynamics simulation data with global distance test (GDT-TS) values (calculated on the Cα atoms), global distance test main-chain prediction (GDT-HA), along with corresponding root mean square deviation (RMSD) with MolProbity.

S. No	Model	3Drefine Score	GDT-TS	GDT-HA	RMSD (Å)	MolProbity
1	2	4160.69	1.0000	1.0000	0.172	−3264.862351
2	3	4111.35	1.0000	1.0000	0.194	−3257.601994
3	1	4716.22	1.0000	1.0000	0.135	−3230.220671
4	4	4056.68	1.0000	1.0000	0.222	−3216.237507
5	5	4005.46	1.0000	1.0000	0.248	−3202.466724

**Table 2:** CABS-flex server results of tau with 12 simulated models along with the C^α^ RMSD scores between predicted models. The least RMSD model was considered to be the best refined model for further analysis.

Models	1	2	3	4	5	6	7	8	9	10	11	12
1	0.00	1.81	3.26	2.46	3.20	4.95	3.86	5.88	4.14	6.51	4.33	6.47
2	1.81	0.00	3.59	2.71	3.30	5.21	3.88	5.91	5.16	6.39	5.08	6.71
3	3.26	3.59	0.00	2.82	2.84	5.11	3.87	6.57	4.88	6.83	4.25	7.10
4	2.46	2.71	2.82	0.00	2.81	4.58	3.55	6.21	4.25	6.53	3.60	6.45
5	3.20	3.30	2.84	2.81	0.00	6.06	5.01	6.22	5.01	6.90	4.55	6.19
6	4.95	5.21	5.11	4.58	6.06	0.00	2.47	4.20	5.41	4.31	5.05	5.43
7	3.86	3.88	3.87	3.55	5.01	2.47	0.00	4.91	5.21	4.51	4.78	6.38
**8**	5.88	5.91	6.57	6.21	6.22	4.20	4.91	0.00	7.03	3.57	6.99	3.26
**9**	4.14	5.16	4.88	4.25	5.01	5.41	5.21	7.03	0.00	7.53	2.99	7.15
**10**	6.51	6.39	6.83	6.53	6.90	4.31	4.51	3.57	7.53	0.00	7.18	5.13
**11**	4.33	5.08	4.25	3.60	4.55	5.05	4.78	6.99	2.99	7.18	0.00	6.91
**12**	6.47	6.71	7.10	6.45	6.19	5.43	6.38	3.26	7.15	5.13	6.91	0.00

**Table 3:** The CABS-flex results of the p-tau protein simulated models table containing RMSD and GDT_TS values (calculated on the Cα atoms) between the predicted models. Model 1 had the least RMSD with 6.94 and was considered the best model for further docking analysis.

Models	1	2	3	4	5	6	7	8	9	10	11	12
RMSD	6.94	7.74	7.21	7.70	7.26	8.81	8.69	8.53	6.79	9.47	7.33	8.10
GDT_TS	0.41	0.38	0.39	0.38	0.39	0.39	0.38	0.33	0.45	0.30	0.43	0.32

**Table 4:** Docking table with bonding characterization and binding energies in kcal/mol with top five therapeutic ligands involved in p-tau inhibition.

S. No	Compound	3-D Structure	Docking Score (S)	Binding Energy (kcal/mol^−1^)	Binding Affinity	Bonding Interaction	Bond Length (Å)	Bond Type
1	Ligand 1	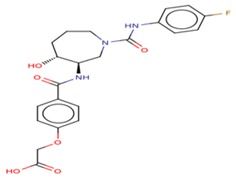	−11.39	−110.32	2.6	Asn 20 Ser 23 (S285)	2.2 2.4	H-acc H-don
2	Ligand 2	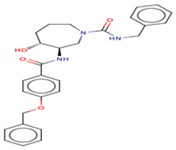	−10.73	−110.43	2.2	Ser 23 (S285) Gln 3	2.2 2.5	Ionic Ionic
3	Ligand 3	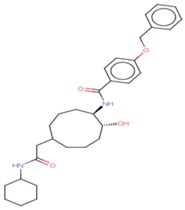	−10.45	−100.66	2.8	Gln 22 Ser 23	1.8 2.2	Ionic Ionic
4	Ligand 4	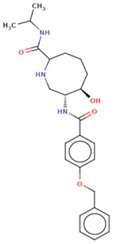	−10.39	−123.36	2.7	Ser 19 Asn 20 Ser 23 (S285)	2.7 2.7 2.2	H-don H-acc Ionic
5	Ligand 5	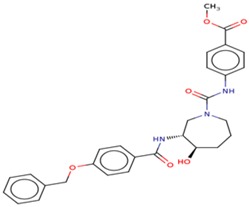	−10.10	−111.03	1.6	Gly 22 Ser 23 (S285)	2.0 2.3	Ionic Ionic

**Table 5:** Blood brain barrier (BBB) penetration analysis with a lazar tool prediction for top five therapeutic ligands involved in p-tau inhibition.

S. No	Compound	Structure	Blood Brain Barrier Penetration (Human) Prediction	Probability Penetrating	Probability Non-Penetrating
1	Ligand 1	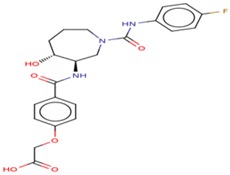	Penetrating	0.0944	0.0722
2	Ligand 2	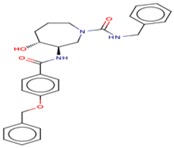	Penetrating	0.15	0.0667
3	Ligand 3	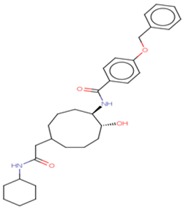	Penetrating	0.157	0.0933
4	Ligand 4	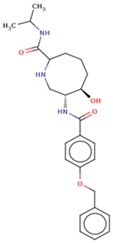	Penetrating	0.137	0.0738
5	Ligand 5	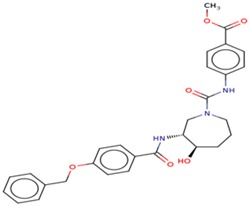	Penetrating	0.141	0.0539

**Table 6:** (**a**) FAF-Drugs4 results tabulated for top five successive ligand small molecules; (**b**) admetSAR toxicity results of top five successive ligand small molecules of virtual ligand screening data.

a. FAF-drugs4						
S. No	Property	Ligand 1	Ligand 2	Ligand 3	Ligand 4	Ligand 5
1	Molecular formula	C22H24FN3O6	C28H32N3O4	C31H42N2O4	C25H33N3O4	C29H31N3O6
2	Molecular weight	445.44	473.56	506.68	439.55	517.57
3	Number of HBA	9	**7**	**6**	7	9
4	Number of HBD	4	3	3	4	3
5	Mol *Log P*	1.69	3.42	5.71	2.71	3.34
6	Related topological surface area (tPSA) A^2^	131.03	90.90	87.66	104.27	117.20
**b. admetSAR**						
1	Human Intestinal Absorption	HIA+	HIA+	HIA+	HIA+	HIA+
2.	Human Ether-a-go-go-Related Gene Inhibition	Weak inhibitor	Weak inhibitor	Weak inhibitor	Weak inhibitor	Weak inhibitor
3	AMES Toxicity	Non AMES toxic	Non AMES toxic	Non AMES toxic	Non AMES toxic	Non AMES toxic
4	Carcinogens	Non-carcinogens	Non-carcinogens	Non-carcinogens	Non-carcinogens	Non-carcinogens
5	Fish Toxicity	High FHMT	High FHMT	High FHMT	High FHMT	High FHMT
6	Acute Oral Toxicity	III	III	III	III	III
7	Aqueous solubility	−3.3390	−3.4553	−3.9841	−3.3390	−3.5326

HBA = hydrogen bond acceptors, HBD = hydrogen bond donors, AMES = Mutagens identified via toxicity test, FHMT = Fish toxicity.
